# El largo y sinuoso camino en la comprensión de la insuficiencia cardíaca

**DOI:** 10.47487/apcyccv.v1i3.79

**Published:** 2020-09-30

**Authors:** Manuel Chacón-Diaz

**Affiliations:** 1 Editor General APCyCCV Servicio de cardiología clínica, Instituto Nacional Cardiovascular INCOR-EsSalud; Lima, Perú Servicio de cardiología clínica Instituto Nacional Cardiovascular INCOR-EsSalud Lima Perú

**Figura 1:**
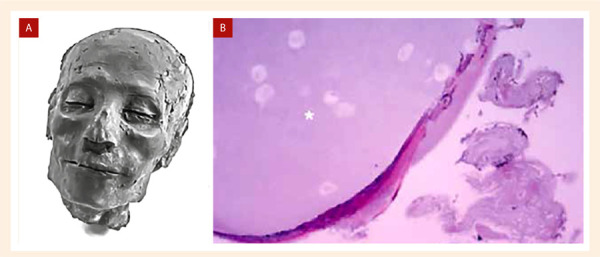
A) Momia de Nebiri. B) Microfotografía pulmonar que muestra edema intraalveolar (asterisco) en el pulmón de Nebiri (Tinción: H/E, magnificación x600) ^1^

La insuficiencia cardiaca (IC) es, hoy en día, una de las más importantes causas de morbimortalidad a nivel mundial, pero no es una entidad nueva para la humanidad. Existen datos ^1^ de la presencia de esta enfermedad desde hace aproximadamente 3500 años, según el análisis de restos de una momia descubierta en el Valle de las Reinas en Egipto pertenecientes a un dignatario egipcio llamado Nebiri, que habría fallecido por «edema pulmonar» de origen cardiaco (Figura 1**).**

**Figura 2:**
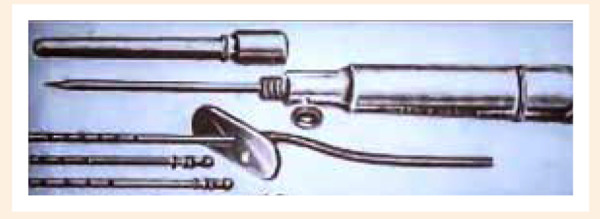
Tubos de Southey ^2^

Durante los siguientes 35 siglos de historia, poco o nada se hizo por comprender esta enfermedad, hubo algunos intentos como las descripciones de «hinchazones hidrópicas» en China (2600 AC) o la creencia de que el corazón servía para producir calor (Galeno, 200 DC). No fue hasta el año 1628 de nuestra era, cuando William Harvey describe la circulación, creando así las bases para la teoría hemodinámica de la IC. Los médicos de entonces; usando solo la semiología, describieron hallazgos como soplos o la dilatación cardiaca (que se asumía debilitaba la fuerza del corazón) y solo se consideraba que el tratamiento era eliminar el fluido retenido, obviamente no existían los diuréticos y usaban dispositivos como los tubos de Southey, que se colocaban en las zonas edematosas periféricas para «drenar» el líquido **(**Figura 2**)**; así mismo, eran frecuentes las sangrías o el uso de sanguijuelas con el mismo fin, hasta que en 1785 William Withering comenzó a usar un producto derivado del reino vegetal que se conocería como digital ^2^.

Tres momentos definirían el rumbo de la medicina cardiovascular: el descubrimiento de los rayos X por Roentgen y del electrocardiograma por Einthoven a finales del siglo XIX que mejoraron el estudio de la IC y la descripción por E.H. Starling en 1918 de la «ley del corazón» que contradecía lo que era asumido hasta entonces como un dogma, mencionando que la dilatación (aumento de volumen diastólico) del ventrículo izquierdo aumentaba su fuerza contráctil y no la debilitaba ^3^. Hasta antes de la década de 1980 ya contábamos con diuréticos (primero las tiazidas en 1958) y la digoxina para el manejo de la IC, los inotrópicos comenzaron a usarse en los años sesenta; E. Braunwald, en 1962, acuña el término fracción de eyección a través del estudio de radioisótopos y, por supuesto, se realizó el primer trasplante cardiaco (C. Barnard, 1967). Pero todos estos tratamientos estaban basados en la teoría hemodinámica de la IC, en la que era importante aumentar la contractilidad cardiaca o disminuir el volumen de sobrecarga como metas del tratamiento.

Recién hace menos de 40 años, con el cambio del paradigma hemodinámico al neuroendocrino como causante de la IC, y con la aplicación progresiva de la medicina basada en la evidencia científica, hemos podido apreciar un avance notable en la comprensión y el tratamiento de la enfermedad. Así, el estudio CONSENSUS I (enalapril, 1987) ^4^, CIBIS II (bisoprolol, 1999) ^5^ y RALES (espironolactona, 1999) ^6^ marcaron el inicio de varios estudios multicéntricos, randomizados, sobre el uso de inhibidores del sistema renina-angiotensina-aldosterona, betabloquadores y antagonistas de aldosterona, respectivamente, los cuales han sido la piedra angular del tratamiento de la IC, sin dejar de lado a los diuréticos, tratamiento sintomático que sin tener la «gran evidencia» de estudios randomizados, han sido la base del tratamiento de la congestión, particularmente en la fase aguda o descompensada de la enfermedad.

Los primeros años del siglo XXI vieron el nacimiento de los dispositivos intracardiacos (cardiodesfibrilador implantable, resincronización) para disminuir la mortalidad y los síntomas de IC (SCDHeFT, 2005 y MIRACLE, 2002) ^7^^,^^8^ lo que aumentó una línea de tratamiento importante para esta entidad. El avance de la investigación médica no se detuvo y, en los últimos años, el descubrimiento de nuevas vías fisiopatológicas en la IC, permitió que el uso de sacubitrilo/valsartan (PARADIGM, 2014) ^9^ , de los inhibidores del cotransportador sodio-glucosa 2 (DAPA-HF,2019 y EMPEROR-REDUCED, 2020) ^10^^,^^11^ y de estimulantes de la guanilato ciclasa (VICTORIA, 2020) ^12^, hayan logrado posicionarse como fármacos de primera línea (al menos los dos primeros) para disminuir la mortalidad y las hospitalizaciones de pacientes con IC con fracción de eyección reducida (ICFEr).

Por otro lado, el reconocimiento a finales de los años 90, de pacientes con síntomas de IC, pero con fracción de eyección normal, generó una serie de investigaciones que, inicialmente, acuñaron el término «IC diastólica», y que a raíz del estudio CHARM preserved (2003) ^13^^)^ se modificó al término de IC con fracción de eyección preservada (ICFEp); ello nos presentó una paradoja de diagnóstico (ya que la IC requería hasta entonces la disminución de función sistólica de ventrículo izquierdo), donde los síntomas de falla cardiaca se originaban por una disfunción periférica primaria (hipertensión arterial, obesidad, etc.). A pesar del avance en los tratamientos para ICFEr, ICFEp no ha podido ser adecuadamente enfrentada ni tratada, tal como lo menciona Saldarriaga *et al*. ^14^ en el número anterior de esta revista. Tal vez llegó el momento de dejar de usar el término ICFEp y desglosarla entre sus diferentes fenotipos y fisiopatología, que no pueden ser englobadas en una, como en el caso de ICFER; así, nuevos estudios podrían ofrecernos terapéuticas adecuadas que impacten en puntos duros… ¿quién sabe?

**Figura 3:**
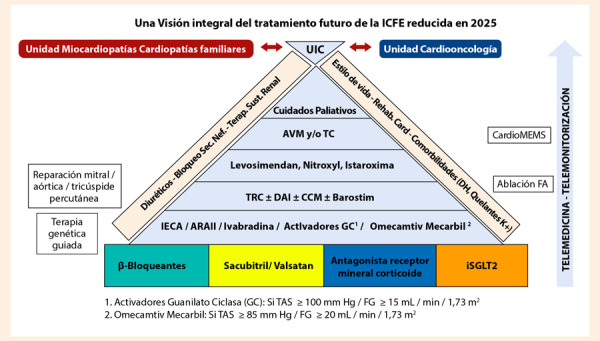
Estrategias terapéuticas en el tratamiento de la IC al 2025

Lo que nos depara el futuro en IC probablemente pasará por cambiar nuestra percepción de clasificar la IC en función de la fracción de eyección (que dicho sea de paso no tiene un criterio único y consensuado de «normalidad»), el mayor uso de dispositivos remotos de monitoreo como cardioMEMS, terapia genética, inteligencia artificial y el crecimiento y fortalecimiento cada vez mayor de las unidades de IC, que todavía es una tarea pendiente en nuestro país. En este punto cito al Dr. Nicolás Manito (@Dr_Manito) y su pirámide de tratamiento, que nos ofrece una visión al 2025 del manejo de la IC **(**Figura 3**)**.

UIC: unidad de insuficiencia cardiaca. AVM: asistencia ventricular mecánica. TC: trasplante cardiaco. TRC: terapia de resincronización cardiaca. DAI: desfibrilador automático implantable. CCM: moduladores de contractilidad cardiaca. Fuente: Reproducido con licencia de @Dr_Manito

En este contexto, les presentamos el tercer número de nuestra revista, en el cual la Dra. Morejón en su artículo: **«Resistencia diurética e insuficiencia cardiaca: entre la congestión y la disfunción renal»,** nos da una brillante explicación de la interacción corazón-riñón en la IC descompensada, planteando estrategias para vencer la resistencia diurética y cambiar la costumbre de basarnos en la creatinina como el mejor marcador de la función renal y predictor único de la respuesta a diuréticos.

Rodríguez *et al.* en: **«Actualización en insuficiencia mitral funcional: una revisión integral»**, revisan el compromiso de la válvula mitral secundaria a la patología miocárdica propia del paciente con ICFEr y nos explica los mecanismos de origen más aceptados y sus alternativas terapéuticas.

Baltodano *et al.*, en su artículo original: **«Deformación miocárdica evaluada por ecocardiografia bidimensional en pacientes lúpicos de un hospital nacional»**, presentan el uso de esta técnica de imágenes en el diagnóstico precoz del compromiso sistólico, en pacientes con lupus eritematoso sistémico en el Hospital Guillermo Almenara de Lima.

Pitta *et al.* presentan: **«Experiencia de 5 años en soporte circulatorio mecánico de corta duración en pacientes post infarto de miocardio: Un reporte del registro INCORMACS»** y nos muestran los primeros resultados del uso de estos dispositivos de corta duración en nuestro hospital.

Por último, Polo *et al.* nos muestran las diversas facetas en la presentación, compromiso cardíaco y tratamiento de la endocarditis infecciosa complicada en nuestro centro en su artículo: **«Experiencia de cinco años en el manejo de endocarditis infecciosa complicada en un centro de referencia nacional».**

Los miembros del consejo editorial de Archivos Peruanos de Cardiología y Cirugía Cardiovascular esperamos que los artículos de este número sean de su interés.
